# Changes in Healthcare Provision During Covid-19 and Their Impact on Children With Chronic Illness: A Scoping Review

**DOI:** 10.1177/00469580221081445

**Published:** 2022-03-28

**Authors:** Sapfo Lignou, Jenny Greenwood, Mark Sheehan, Ingrid Wolfe

**Affiliations:** 1 Nuffield Department of Population Health, Ethox Centre and Wellcome Centre for Ethics and Humanities, 6396 University of Oxford, Oxford, UK; 2 Kings Health Partners, 4616 King’s College London, London, UK; 3 Nuffield Department of Population Health, Ethox Centre, 6396 University of Oxford, Oxford, UK; 4 4616 King’s College London, London UK

**Keywords:** telemedicine, paediatrics, health systems, covid-19, chronic illness, policy, mental health, child healthcare services

## Abstract

This paper provides an overview of the evidence around how the health systems and policy response to the Covid-19 pandemic affected children with long-term conditions in the UK. We conducted a scoping review guided by the PRISMA-ScR Checklist. The PubMed and PsycINFO databases (2019-August 2021) were searched and screened for papers (of any design) by 2 reviewers independently. The electronic database search was supplemented by manual searching. A total of 32 papers were identified, including studies on UK paediatric populations, studies on chronic illness in the UK, and international studies on chronic illness and children (including data from the UK). Most studies focussed on epilepsy, cancer, diabetes or asthma. Three categories of impact were identified: (*a*) impact of policy response on the delivery of and access to child healthcare (*b*) impact of innovative practice on children’s physical and mental health (*c*) impact of service restrictions on children’s physical health. Our results showed that policy response to the pandemic significantly affected healthcare provision for children with chronic illness in the UK. However, the specific assessment of the impact of service restrictions and innovative practice on children’s health and wellbeing is limited. Future research is required to fill knowledge gaps on changes in access to effective diagnostic and treatment investigations and their impact on a range of paediatric patients during the pandemic.

## Introduction

Children with chronic illness and long-term conditions require both planned and urgent care, over long periods of time. The quality of this care depends on established therapeutic relationships and the interaction across health services, such as inpatient and outpatient health and social care services.^1^ The multiple and complex needs that characterise this group of children also renders them particularly vulnerable to changes in the delivery of care.

Pandemic restrictions were introduced in the UK on 23rd March including social distancing and shielding for those with acute physical conditions.^2,3^ National Health Services (NHS) around the country adjusted healthcare delivery policies, affecting all populations, and children in specific ways. Many Tier 1 paediatric staff were redeployed and paediatric inpatient space was lost to adult services^4^. Based on a nationwide survey by the Royal College of Paediatrics and Child Health (RCPCH), up to 90% of services reported a decrease in inpatient activity at the beginning of April, falling to 49% by 3rd July. Only 9% reported an increase in inpatient activity compared to the same time last year.^4^

The effects of the pandemic are expected to dominate healthcare delivery in the UK for at least the next few years.^5^ It is therefore crucial to understand the nature and degree of impact of health system changes on children with long-term conditions.

This scoping review aims to explore the currently available evidence on how the health systems policy response to Covid-19 has impacted children with chronic conditions in the UK.

## Methods

As our purpose was to identify and map emerging evidence in a fast changing situation regardless of study design, a scoping review rather than a systematic review was preferable.^6^ The review was conducted according to the PRISMA-ScR.^7^ The research was formulated around the PICO model: the Population of interest was children with chronic conditions; the Intervention was the emergence of the Covid-19 virus and subsequent changes to healthcare provision, which were Compared with pre-pandemic standard of care. We examine the impact and outcomes of these changes to healthcare provision on children with chronic conditions.

### Search Strategy

The scoping review was guided by the following question: ‘How have health systems policy responses to Covid-19 affected children with chronic illness in the UK?’ Two databases, PubMed and PsycINFO, were searched from March 2020 to August 2021. Two queries were used in each database, a broad query focussed on primary care/hospital records and projected health outcomes and a specific query focussed on qualitative research about healthcare professionals and patients/carers views.

Keywords relating to Covid-19, child healthcare, chronic illness and impact were used. The search strategy [Appendix 1] was drafted by SL with the help of an experienced librarian and further refined through team discussion. Limits on number of results and other filters were not set.

### Study Inclusion and Charting of Data

In the first instance, two of the authors (SL and JG) screened the article titles and abstracts independently and selected articles using inclusion criteria [supplementary material] to account for any impact reported on Chronic Health Conditions (CHC) in the UK during the pandemic. The remaining articles were then screened in full by the 2 authors independently. Articles were selected based on the following criteria. Inclusion criteria are as follows: UK studies on Covid-19 and paediatric population, UK studies on Covid-19 and patients with chronic illness, UK studies on Covid-19 and health services, international studies including data from the UK on Covid-19 and chronic illness. Exclusion criteria: UK studies not related to Covid-19 and chronic illness or health systems, international studies on adult and child populations and Covid-19 not related to chronic illness, international studies on paediatric chronic illness and Covid-19 not referring to the UK.

Disagreements on study selection and data extraction were resolved by consensus and discussion with the other authors (MS and IW) when required.

The electronic database search was supplemented with manual searching. Reference lists of the final articles from the database search were hand-searched by SL and JG and relevant papers included. Grey literature sources, including academic websites and websites of UK professional medical bodies (e.g. the Royal College of Paediatrics and Child Health), identified during the search period were also searched in order to ensure comprehensiveness. This manual search identified key grey literature sources and online reports which were not included in database searches.

A data-charting form was jointly developed by SL and JG to determine which variables to extract. The 2 authors independently charted the data, discussed the results and continuously updated the data-charting form in an iterative process.

Data were abstracted based on article aims and objectives, population (e.g. adult and child population, UK paediatric and international paediatric), health condition examined (e.g. asthma and mental health), healthcare type (e.g. unplanned/emergency hospital admissions and primary care), and main findings for type of impact assessed.

A summary table demonstrating characteristics of the final articles is included in Table 1.

**Table 1.** Descriptive Analysis of the Included Articles (Sorted in Alphabetical Order)

Author, Year	Setting	Constituent countries	Study design	Study population	Sample size	Age range	Type of chronic illness	Type of impact/impact theme	Major outcomes
Ashikkali, Carroll & Johnson (2020)	Emergency and routine care	UK	Literature review	UK, paediatric	N/A	Unspecified paediatric population (0-18)	Physical and mental health conditions	A4, C1	There was a decrease of over 30% in the cases of children presenting to the paediatric emergency department by March 2020 which was maintained into the summer. This may have meant reduced unnecessary attendance to the department however it a lack of treatment could also put children with serious pathologies at greater risk.
Ashton, Batra & Coelho et al. (2020)	Paediatric Gastroenterology	UK, China, South Korea	Literature review	International, paediatric	N/A	Unspecified paediatric population (0-18)	Inflammatory bowel disease	A2, A4	Healthcare professionals could not provide the same level of diagnostic care and ongoing management to patients with inflammatory bowel disease during the pandemic.
Ashton, Kammermeier & Spray et al. (2020)	Tertiary paediatric IBD centres	UK	Survey	UK, paediatric	20 tertiary paediatric IBD centres	Unspecified paediatric population (0-18)	Inflammatory bowel disease	A4, C1	Diagnostic IBD practice has been severely impacted by COVID-19. More than 50% of new diagnoses did not include endoscopy.
Cameron, Hauari & Hollingworth et al. (2020)	Routine healthcare	UK	Survey	UK, adults and children	522 adult respondents (members of general public within borough of Tower Hamlets)	Unspecified (child and adult population)	Physical and mental health conditions	C	Covid-19 impacted many areas of child healthcare including newborn screening, developmental checks, immunisations and health visiting. There was an ethnic disparity with White respondents more likely to access reviews and immunisations than other ethnic groups.
Charlesworth, Bold & Pal (2021)	Emergency care	UK	Retrospective observational cohort study	UK, paediatric	Unspecified	0-15	Infective disease, sequelae of infective illness, respiratory disease	C3	Public health measures significantly altered paediatric presentations. Oxfordshire hospitals saw a 58% reduction in ED attendances/inpatient admissions. Missed diagnoses were predominantly seen in infection-related illnesses.
Creese, Taylor-Robinson & Saglani, et al. (2020)	Primary Care	UK	Literature review	UK, paediatric	N/A	Unspecified (child and adult population)	Asthma	C1, C3	There were fewer severe asthma presentations in CYP to emergency room during the pandemic. School closures and social distancing meant fewer triggers from respiratory infection. Caregivers’ heightened concern for children with asthma likely improved adherence to medication and reductions in air and road traffic led to the reduction in some air pollutants across the UK.
Elbarbary, Jeronimo dos Santos & de Beaufort et al. (2020)	Diabetes centres	75 countries. Majority from UK (35; 16.3%), USA (20; 9.3%) and India (15; 7%)	Survey	International, paediatric	303 respondents (healthcare professionals)	0-16	Diabetes	A2, A5	Healthcare professionals adapted to the pandemic by implementing telemedicine. One fourth of professionals reported delays in diagnosis and an increased rate of Diabetic ketoacidosis. The pandemic had an important impact on family’s behaviour that might have led to increase in DKA presentation.
Forde, Arente & Ausili et al. (2021)	Specialist, community or in-patient settings	Belgium; Bosnia and Herzegovina; Croatia; Cyprus; Czechia; Denmark; Estonia; Finland; France; Germany; Greece; Ireland; Italy; Latvia; Malta; Netherlands; Norway; Poland; Portugal; Romania; Spain; Sweden; Switzerland; Turkey; Ukraine; United Kingdom (UK).	Survey	International, adults and children	1829 respondents (diabetes nurses)	Unspecified (child and adult population)	Diabetes	C1, B	There was an increase in both physical and psychological problems in patient populations during the pandemic and clinical diabetes services were significantly disrupted.
Hartmann-Boyce, Morris & Goyder et al. (2020)	Routine healthcare	Multiple, including UK, US, China	Literature review	International, adults and children	N/A	Unspecified (child and adult population)	Diabetes	B	After social distancing and shielding guidance was issued, primary and secondary care began to provide both emergency and routine follow-up on the phone or via video consultations. This also included support for mental health
Hefferon, Taylor & Bennett et al. (2020)	Emergency care and community paediatrics	UK	Literature review	UK, paediatric	N/A	Unspecified paediatric population (0-18)	Acute physical conditions and mental health conditions	A2, A3, A5	Disruptions to planned outpatient visits, operations or healthcare may have led to increased morbidity for some children. These changes have also led to increased anxiety for families.
Isba, Edge & Jenner et al. (2020)	Emergency care	UK	Retrospective cohort study	UK, paediatric	Unspecified	<16	Physical conditions and safeguarding	A3	There was a decline in paediatric emergency department attendances meaning many children may be at home with serious pathologies or illnesses.
Jia, Bao & Yi et al. (2020)	Paediatric respiratory clinics	UK	Interviews	UK, adults and children	16 (caregivers)	Caregivers of children <14	Asthma	C	Six main themes identified: (1) improved asthma control; (2) decreased willingness to seek medical care; (3) increased adherence to treatment; (4) coping strategies for changes caused by the pandemic; (5) a new opportunity and 6) managing new challenges in asthma control.
Kelly & Firth (2020)	Emergency care	UK	Literature review	UK, adults and children	Unspecified	Unspecified	Gastroenteritis, respiratory conditions	C3	On 14 May 2020 A&E visits were 57% lower in the previous month compared with April 2019. A&E visits across all unit types dropped by 57% in April, but there was a greater decrease in minor A&E units than in major A&E units.
Kursumovic, Cook & Vindrola-Padros et al. (2021)	Anaestheia and critical care	UK	Survey	UK, adults and children	470 (healthcare professionals)	Unspecified (child and adult population)	Non-cancer elective, cancer	A4	There was increased systemic pressure on anaesthetic and peri-operative services due to the demand of the pandemic on critical care.
Lynn, Avis & Lenton et al. (2021)	Emergency care and paediatric assessment units	UK	Survey	UK, paediatric	4075 (paediatric consultants)	0-16	Diabetes, sepsis, malignancy	A3, C1	Diabetes mellitus was the most common delayed presentation, as well as sepsis and malignancy. There were nine deaths where delayed presentation was considered a contributing factor, resulting mainly from sepsis and malignancy.
Mansfield, Mathur & Tazare et al. (2021)	Primary care	UK	Statistical analysis	UK, adults and children	Jan 1 2017: 9,863, 903 (population included in analysis) Jan 1 2020: 10, 226,939 (population included in analysis)	11+	Acute physical and mental health conditions	A1	Primary care contacts for almost all conditions examined dropped considerably after Covid-19 restrictions were implemented, particularly for diabetic emergencies, depression and self-harm.
Maringe, Spicer & Morris et al. (2020)	Primary and secondary care	UK	Population-based modelling study	UK, adults and children	93,607 (patients)	Unspecified (child and adult population)	Cancer	A4, A5, B, C2	Between 3291-3621 avoidable deaths would have occurred in 5 years after diagnosis across four tumour types. It is predicted that between 59,204-63,229 years will be lost due to delays in cancer diagnosis alone as a result of the COVID-19 lockdown in the UK.
Mulholland, Wood & Stagg et al. (2020)	Emergency care and emergency and planned hospital admissions	Scotland	Statistical analysis	UK, adults and children	Unspecified	0-85+	Paediatrics (medical), Paediatrics (surgical)	A3	There was a sharp drop in A&E attendances as well as planned hospital visits. Children aged under 15 years were particularly affected by reduced access to emergency care, both in terms of attendances and admissions.
Ogundele & Ayyash (2021)	Community Child Health Services	UK	Literature review and scoping survey	UK, paediatric	62 responses to survey (healthcare professionals)	Unspecified paediatric population (0-18)	Neurodevelopmental/emotional/behavioural disorders	A2, A4	Restrictions in face-to-face contacts and redeployment of staff from Community Child Health (CCH) services meant that many core clinical activities were limited and most new referrals were kept on hold except for urgent services. Many services greatly increased their telemedicine capacity.
Papadopoulos, Custovic & Deschildre et al. (2020)	Paediatric asthma clinics	27 countries from 5 continents; Africa, Asia, Americas, Europe, and Oceania	Survey	International, paediatric	Ninety-one respondents (carers for approximately 133,000 children with asthma)	Unspecified paediatric population (0-18)	Asthma	A4, C1	Outcomes for some children with asthma may have improved, possibly due to increased adherence and/or reduced exposures. Asthma services responded to the pandemic by conducting virtual encounters instead of physical appointments.
Reilly, Muggeridge & Cross (2021)	Routine care	Scotland	Survey	UK, paediatric	201 (young people: 71 and caregivers: 130)	Young people (12-25) Caregivers (0-25)	Epilepsy	B, C1	The pandemic and the associated restrictions have negatively impacted young people with epilepsy. Participants reported increases in seizures, reluctance to go to hospital and cancelled investigations. The wider psychosocial impact includes increases in child and caregiver mental health problems.
Roland, Harwood & Bishop et al. (2020)	Emergency care	UK	Multi-centre surveillance	UK, paediatric	1349 respondents (healthcare professionals)	<16	Soft tissue injuries or fractures but no other specific pathologies	A3, C3	93.5% of parents were felt not to have delayed presentation. Delayed presentation was relevant in 3% of cases. In 8% of cases, advise from a medical professional or NHS 111 was considered to have resulted in delay.
Serlachius, Badawy & Thabrew (2020)	Emergency and routine care	UK, Ireland, Germany, Canada, Australia, China, Italy, Singapore, New Zealand, Austria.	Literature review	International, paediatrics	Unspecified	Unspecified paediatric population (0-18)	Chronic health conditions	B	Challenges identified include: increased anxiety, disrupted routines, academic and social stresses associated with school closure, increased risk of domestic violence and abuse, and reduced access to physical and psychosocial support. Opportunities include: reduced academic and social stress, increased time with families, reduced access to substances, easier access to health care using technology, and opportunities to build resilience.
The Royal College of Paediatrics and Child Health (2020)	Emergency and routine care	UK	Survey	UK, paediatric	30% to 53% respondents per week (healthcare professionals)	Unspecified paediatric population (0-18)	Diabetes, mental health conditions, sepsis	A3, C1, C2	Paediatric care activity across all areas decreased or was unchanged compared to the same week the previous year, causing many healthcare professional to report concern about the wellbeing of the children they weren’t seeing.
Thornton (2020)	Emergency care	UK	Literature review	UK, adults and children	Unspecified	Unspecified (child and adult population)	Pneumonia, cardiac, myocardial ischaemia, and gastrointestinal conditions	C3	In the week after lockdown (March 23-29) attendance at A&E was down 25% on previous week.
Thorpe, Ashby & Hallab et al. (2021)	Specialist care and unplanned/emergency hospital admissions	UK	Survey	UK, adults and children	463 respondents (people with epilepsy: 316 and caregivers on behalf of a person with epilepsy: 147)	<18->65	Epilepsy	A1, A4, B, C3	Responses included reported change in seizures, mental health difficulties and sleep disruption. Participants reported finding it difficult to take medication on time. There were difficulties accessing medical services and cancelled appointments.
Watson, Pickard & Williams et al. (2021)	Primary and secondary care	UK	Semi-structured interviews	UK, paediatric	15 participants (caregivers)	Caregivers (25-60) of children (0-16)	Physical health conditions	A1, B	Delays in seeking care occurred predominantly due to fear, community perception and experience and media portrayal. Reported delays in reaching care were focussed on availability of services and access to primary care.
Williams, Jenkins & Ashcroft et al. (2020)	Primary care	UK	Retrospective cohort study	UK, adults and children	241,458 (patients)	Unspecified (child and adult population)	Common mental health problems, cardiovascular and cerebrovascular disease, type 2 diabetes and cancer	A5, B, C2, C3	Diagnoses of common conditions decreased substantially between March and May 2020. This suggests a large number of patients have undiagnosed conditions.
Williams, Macrae & Swann et al. (2020)	Primary care, emergency department and emergency hospital admissions	Scotland	Retrospective cohort study	UK, paediatric	Unspecified	0-14	Respiratory conditions, neurological conditions injury, poisoning	A1, A3, C1, C2, C3	There was an almost two-thirds reduction in unscheduled primary care during the study period compared with the same weeks in 2016-19 and also a reduction in emergency department attendances.
Wirrell, Grinspan & Knupp et al. (2020)	Paediatric Epilepsy Centres and Child Neurology Centres	49 countries from 6 continents including Asia (40.6%), North America (36.8%)	Survey	International, paediatric	212 respondents (paediatric neurologists)	Unspecified paediatric population (0-18)	Epilepsy	B, C1	Findings included an increase in the use of telemedicine, decreased EEG use, changes in treatments of infantile spasms, and cessation of epilepsy surgery.
Wise (2020)	Emergency care and paediatric assessment units	UK	Survey	UK, paediatric	2433 respondents (paediatric consultants)	Unspecified paediatric population (0-18)	Diabetes, sepsis, malignancy	C1, C2	Delays in attending the emergency department during the pandemic may have contributed to the deaths of nine children.
Young Minds (2020)	Psychological support services	UK	Survey	UK, paediatric	2,036 respondents (young people)	13-25	Mental health	B	26% of young people in the UK with pre-existing mental health problems were not able to get psychological support during the lockdown

*Note.* A = Impact on children’s delivery of and access to healthcare; A1 = Reduction in use of primary care services and difficulties in accessing medication; A2 = Increased use of telemedicine; A3 = Delayed presentation to A&E departments and paediatric assessment units; A4 = Cancelled or suspended investigations and reduced surgical activity; A5 = Delays in diagnosis; B = Impact of innovative practice on children’s physical and mental health; C = Impact of service restrictions on children’s health; C1 = Increased disease severity; C2 = Increased mortality; C3 = Increased emergency and hospital admissions.

A thematic analysis was conducted on all selected papers to identify the key areas of impact on children during the pandemic. A coding protocol for the thematic analysis was developed by the 2 authors (SL and JG). The main findings relating to impact on children’s care, physical health and mental health were identified and coded. The codes were then grouped into the subthemes of impact type. Finally, the findings were iteratively summarised and grouped by SL and JG and discussed with IW and MS until consensus was reached [Table 1].

## Results

**Figure 1. Flow chart.**
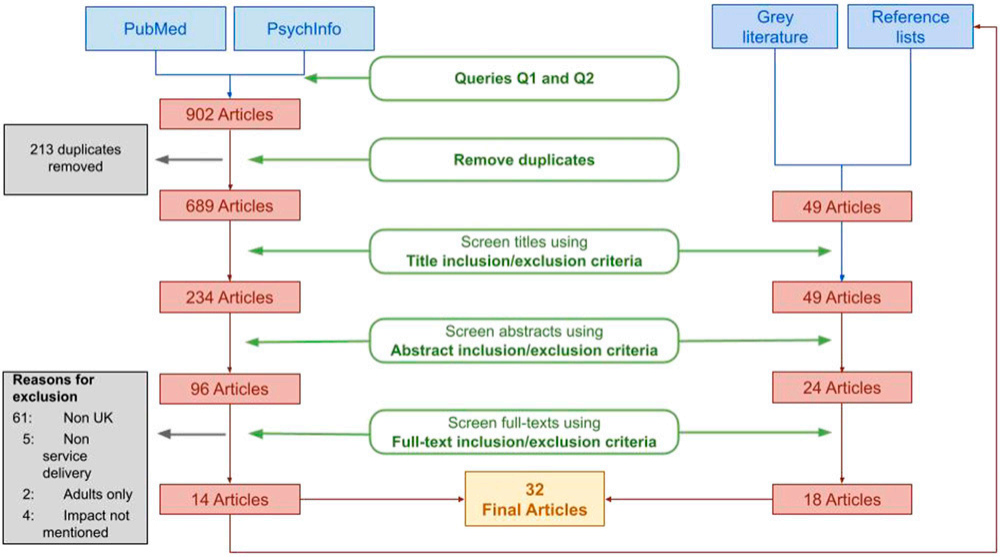

The broad search in PubMed and PsycINFO identified 604 potentially relevant papers across both databases. The qualitative search in PubMed and PsycINFO identified 298 papers across both databases. After duplicates were removed the total number of articles was reduced to 689. Following title and abstract screening the number of selected articles went down to 96. A total of 14 papers were identified after full texts were examined. An additional 18 papers were identified through manual searches and reference lists. In total 32 papers were selected for the analysis [Figure 1].

Only 16 papers specifically focussed on the impact of changes in health care delivery on children in the UK, the others focussed on adult and child population in the UK or child population internationally. Papers directly referring to chronic conditions in children in the UK were limited to specific conditions, as follows: 3 papers focussed on asthma, 3 on diabetes, 3 on epilepsy, 2 on inflammatory bowel disease, 4 on mental health, 1 on neurodevelopment disorders and 1 on cancer. Fifteen papers did not focus on a specific chronic illness, and 9 of these papers explored the general impact of the pandemic on paediatric emergency care. The age range of children involved was from newborns to 18 years old. Most papers examined the impact of changes in healthcare delivery on children’s physical health except Serlachious et al (2020), Williams et al (2020) and Mansfield et al (2021) who discussed psychosocial challenges and impact on children with mental illness.^3,8,9^

### Key Themes

Three themes were identified: (*A*) impact of health systems policy response on the delivery of and access to healthcare (*B*) impact of innovative practice on children’s physical and mental health (*C*) impact of service restrictions on children’s physical health.

### Subthemes Emerged From Theme (A) and Theme (C)

Theme (A): ‘Impact on children’s delivery of and access to healthcare’ was grouped into 5 subthemes: (A1) reduction in use of primary care services and difficulties in accessing medication, (A2) increased use of telemedicine, (A3) delayed presentation to Accident and Emergency (A&E) departments and paediatric assessment units, (A4) cancelled or suspended investigations and reduced surgical activity and (A5) delays in diagnosis.

Theme (C): ‘Impact of service restrictions on children’s health’ was grouped into 3 subthemes: (C1) increased disease severity, (C2) increased mortality and (C3) increased emergency and hospital admissions.

The types of impact described are summarised below.

### Impact on Delivery of and Access to Healthcare

A1 Reduction in use of primary care services and difficulties in accessing medication primary care contacts changed during the first part of the pandemic for children with acute physical and mental health conditions, such as depression and anxiety, eating disorders, self-harm behaviours and obsessive-compulsive disorder, cardiovascular and diabetic emergencies and asthma.^3^ GP contact levels between March and July 2020 were below the average of the previous 3 years, particularly for diabetic emergencies, depression and self-harm.^3^ There was an almost two-thirds reduction in unscheduled primary care visits in Scotland between 23rd March and 9th August 2020 compared with the same weeks in 2016-19.^10^ Some parents in London reported difficulty getting through to the receptionist at their GP surgery.^11^ Many young people with epilepsy and their caregivers reported barriers in acquiring a repeat prescription and contacting primary healthcare physicians or pharmacists.^12,13^

### A2 Increased Use of Telemedicine

There was increased use of telemedicine within universal children’s services such as health visiting.^2,14^ A study on children with neurodevelopmental conditions found restrictions to direct face-to-face clinician contacts, with many Community Child Health (CCH) services increasing their use of telemedicine markedly. Telephone consultation was the most commonly used method of remote contact, reported by 98% of healthcare practitioners.^15^

There was an increase in resources designed for children and young people (CYP) with Emotional, Behavioural, Neurodevelopmental and Intellectual disorders (EBNDID) including Attention Deficit Hyperactivity Disorder (ADHD), Autism, Epilepsy and Cerebral palsy and their families including webinars, online videos, sleep tips and post-diagnosis support.^15^ Video consultations were also increasingly used for children with epilepsy.^12,16^

Telemedicine was also reported as the standard for treatment of children with inflammatory bowel disease during the pandemic and for the majority of children with diabetes.^17-19^

### A3 Delayed Presentation to A&E Departments and Paediatric Assessment Units

Since the start of the pandemic, there has been a significant decrease in under 16-year-olds presenting at and being admitted into A&E and paediatric emergency departments.^4,10,14,20,21^ Lynn et al. (2021) found that 32% of paediatricians working in emergency departments and assessment units reported delayed presentations with a range of between 14% in Wales to 47% in the Midlands.^22^ Two hospitals in Greater Manchester also reported a decline in paediatric emergency department (PED) attendances.^20^ In contrast, according to Roland et al. (2020), who examined hospital presentations with intermediate-risk and high-risk symptoms between 27th April and 15th May 2020, late presentation to emergency departments during this period was rare.^23^

Delayed presentations occurred most commonly in diabetes mellitus (DM) or diabetic ketoacidosis (DKA), along with sepsis and malignancy.^22^ In particular, a third of UK A&E paediatricians reported witnessing delayed presentations for new diagnoses of DM and DKA.^2,3^

### A4 Cancelled or Suspended Investigations and Reduced Surgical Activity

Between the 18th and 31st January 2021, paediatric and non-cancer elective surgical activity was occurring at less than a third of the rate of the previous year. In the most overwhelmed parts of the country, paediatric surgery fell to 12–20% of normal activity.^24^ These reductions significantly impacted the care of children with epilepsy. Between April – Sept 2020 surgical activity for epilepsy was limited or stopped entirely.^12^

Changes in the care of children with epilepsy during the pandemic includes decreased use of Electroencephalogram (EEG) and other cancelled investigations.^12^ Many young people and caregivers in the UK had investigations (EEG or MRI) cancelled by the hospital while some young people and caregivers cancelled investigations themselves.^13^ Video-EEG monitoring and other elective admissions were suspended.^13^

After recommendations by professional bodies and commissioners, multiple changes to cancer care have been established since the start of the pandemic, from the point of diagnosis (e.g. suspension of screening services) to treatment plans.^25^

There was a significant decrease in availability of lung function testing for children with chronic respiratory diseases; however, concerns have been partially overcome in some places with provision of home testing with either peak flow metres or portable spirometers.^14^

A study on children with neurodevelopmental conditions found restrictions to direct face-to-face clinician contacts and redeployment of staff from CCH services. Key clinical activities were limited, non-urgent new referrals were put on hold and CCH teams experienced significantly increased waiting lists.^15^

For children with inflammatory bowel disease there were difficulties in continuing day-case infusions, absence of face-to-face clinics and problems reviewing patients or performing routine blood or stool monitoring.^17,26^ Over 50% of CYP presenting with suspected IBD were diagnosed without the usual histological and endoscopic assessment because of the reduced use of endoscopy at over 90% of centres across the UK.^17,26^ According to 1 study only 13 of the 17 sites with available urgent endoscopy had performed the procedure, which translates to between 8 and 24 patients per site per week.^26.^Usually large paediatric gastroenterology centres would have 2–4 lists per week, with 4–6 patients per list.^26^ Reductions in endoscopy services were compounded by the redeployment of anaesthetic teams, while reduced space, extensive time for cleaning between cases and the need for PPE also affected the provision of these services.^26^

### A5 Delays in Diagnosis

Data collected from Salford in the UK found a large decrease in the rate of new diagnoses for circulatory system diseases, type 2 diabetes, malignant cancers and common mental health problems.^9^ Another UK-based study supported these results for cancer patients. Screening services were suspended and there was an 80% decrease in 2-week wait cancer referrals since March 2020 due to reduced diagnostic services including endoscopies, social distancing rules (including instructions for the public to present at GPs with urgent concerns only) and public health anxiety.^25^

Delayed diagnosis of paediatric DM and DKA have also been reported^2^. This is concerning because DKA is a severe and life-threatening complication of diabetes and for treatment to start as soon as possible, early diagnosis of type 1 diabetes is necessary.^19^ Delays in diagnosis suggest a large backlog of patients may require attention by primary and secondary care.^10^

### Impact of Innovative Practice on Children’s Mental and Physical Health

Digital healthcare increased access for children with mental health difficulties.^8^ 26% of young people in the UK with pre-existing mental health problems were unable to access psychological support during the lockdown; however, a large number reported receiving treatment on the phone or through video calls.^27^ Primary and secondary care services provided both emergency and routine care to people with diabetes on the phone or via video consultations, including mental health support.^28^ Although it is not yet clear what impact the change to telehealth will have on diabetes outcomes, the majority of respondents to a pre-pandemic survey found virtual appointments useful.^18^ In contrast, the use of telehealth for people with cancer suggests a greater proportion of missed diagnoses.^25^

Use of telemedicine also increased for children with epilepsy.^12^ Virtual diagnosis without the use of EEG could have decreased accuracy, and misdiagnosis could have adverse outcomes for children unnecessarily exposed to the side effects of treatment.^16^ Additionally, having difficult health-related conversations virtually may negatively impact patient mental health.^16^ The majority of respondents in one study considered telehealth to be as effective as in-person consultations.^12^ However, some young people with epilepsy and their caregivers stated that they were less satisfied with telehealth.^12^ Many respondents to another survey felt that the care received through telehealth with the GP was inadequate, with language barriers cited as a factor.^11^ In-person appointments were preferable for patients with additional needs, such as those with autism or hearing loss.^13^

Reliance on telehealth may have resulted in missed diagnoses for common mental health problems, cardiovascular and cerebrovascular disease, type 2 diabetes and cancer.^9^ Delays in diagnosis for these conditions have been associated with higher rates of mortality, with particular concern for patients with depression.^9^

### Impact of Service Restrictions on Children’s Physical Health

#### C1 Increased disease severity

Decreases in primary care visits, A&E attendances and unplanned hospital admissions did not result in greater disease severity upon presentation to PICU in Scotland during the lockdown.^10^ However, other research suggests that reduced access to healthcare and disruptions to planned outpatient visits and operations may have led to increased child morbidity.^2,4^ This is a particular concern for children who are also missing the developmental support and access to therapies from school, such as children with cerebral palsy or musculoskeletal problems.^14^ Many health professionals were concerned about the wellbeing of the children they were not seeing.^4^

A prominent concern was about increased seizure severity in children with epilepsy due to difficulties accessing medicine, reduced access to health professionals and the additional stress, mood changes and sleep problems associated with the pandemic.^12,13^ Additionally, the developmental trajectory of children with developmental and epileptic encephalopathies is negatively affected by delayed or ineffective treatment.^16^ Some children will suffer irreparable neurodevelopmental harm or even premature morbidity as a consequence of delays in epilepsy surgery evaluations.^16^

The health of children with diabetes has also been significantly affected by healthcare delivery changes during the pandemic.^18^ A study on the views of diabetes nurses across Europe found that an increase in acute hyperglycaemia was reported by approximately 50% of respondents, with the UK respondents rating physical impact on patients higher compared to other European countries.^18^

Healthcare professionals expressed concerns about the provision of care for children with Inflammatory Bowel Disease (IBD) during the pandemic, particularly the commencement of maintenance systemic immunosuppression without endoscopic or histological diagnosis.^26^

The indirect impact of the pandemic was also significant for cancer patients. For optimal outcomes, timely diagnosis and treatment are vital but services were severely affected. Oncologists were worried about the decreased referral rate for suspected cancer in children.^22,29^

However, not all chronic illnesses have been affected in the same way, with lockdown measures positively affecting many children with asthma. This is notable considering that the UK has one of the highest rates of asthma deaths in Europe.^30^ Adherence to medical routines was improved in many cases, as children spent more time at home with their parents or caregivers.^10,30^ Reductions in air and road traffic, decreased interaction between children, restricted travel, shielding and social distancing may have also contributed. An international study on paediatric asthma reached the same conclusions.^27^

#### C2 Increased mortality

Delayed presentation of children to A&E departments may have resulted in avoidable child mortality and morbidity.^4,29^ Data from Salford showed delayed or missed diagnosis of common mental health problems, cardiovascular and cerebrovascular disease, type 2 diabetes, and cancer in both adults and children, which could have a clinically significant impact on long-term health and mortality.^9^ A further study demonstrated that a decrease in primary care visits, A&E attendances and unplanned hospital admissions were not associated with increased mortality rates in ages 0–14 years^9^. Moreover, despite the increase in PICU admissions, there were no significant changes in paediatric mortality for the period between 29th March and 9th August across any age group examined.^10^

A study on the impact of delays in cancer diagnosis in adults and children estimated that between 3291 and 3621 avoidable deaths will have occurred from 5 cancer types in the 5 years after diagnosis compared with the pre-pandemic period. An additional 59,204–63,229 years of life lost will be attributable to delays in cancer diagnosis alone as a result of the first COVID-19 lockdown in the UK.^25^

#### C3 Increased emergency and hospital admissions

Encouragingly, one study found that among the children with delayed presentations to A&E departments, only 11.8% were admitted to hospital, suggesting limited effect on outcomes.^23^ Contrastingly, Williams et al. (2021) found an increase in symptom severity upon presentation at A&E during the pandemic in Scotland.^10^ Paediatric ED admissions in Oxfordshire were significantly reduced compared with the previous 5 years across all age groups.^31^ Yet despite a decrease in admissions, a greater proportion of children admitted received more than 10 diagnoses, suggesting an increase in children with severe or complex disease.^31^

Fewer children and young people presented at emergency departments with severe asthma during the pandemic.^30,31^ A study on both UK adults and children showed that in the week after lockdown (23rd–29th March) attendance at A&E was down 25% on the previous week.^32^ There was a 77% decrease in admissions to PICU for disorders of the respiratory system and a decrease in admissions for the neurological system according to primary and secondary care providers in Scotland.^10^

In contrast, high numbers of patients were requiring emergency care due to epilepsy or related injuries during the pandemic.^13^ There were more urgent admissions for patients with malignancies, which could be explained by reductions in face-to-face primary care contacts, and/or changes to tertiary oncology services with the restrictions to elective admissions.^31^

## Discussion

The results of this review indicate that delivery of, and access to, healthcare for children with chronic illness was significantly affected during the pandemic. A number of factors led to these changes. These include lockdown measures, the shielding of people with certain health conditions and the recommendations by professional bodies and commissioners prioritising the response to the pandemic.^2,3,8,33^ Similar challenges in the provision of healthcare for children with chronic illness including epilepsy, asthma and diabetes were also reported internationally including in US, Asia and Europe.^16,18,19,34^ Due to the restrictive measures and increased health anxiety, the health-seeking behaviour of UK caregivers changed, with many families deciding to stay away from hospital, to delay presentation at a healthcare setting or to miss their routine health checks.^11,12,35^ Decreases in access to healthcare were seen across healthcare settings including primary care hospital and A&E.^3,10,22,29,36^

Due to the changes in child healthcare provision, significant unmet need was identified which could result in increases in morbidity and mortality for children with various physical and mental health conditions.^2,3^ Social distancing measures meant that vulnerable children missed regular contact with education, health and social care professionals. It is estimated that many children may have been experiencing maltreatment, neglect or domestic violence unknown to professionals.^2,20^ Parents’ reluctance to seek healthcare during the pandemic indicates that many children remained at home with serious pathologies or illnesses.^2^

One of the most prominent changes in the delivery of healthcare across different health conditions was the use of telehealth.^19^ Although this reduced the risk of Covid-19 infection, views on its efficacy are mixed. Inconsistent efficacy rates are expected to lead to a large backlog of patients who require care for undiagnosed conditions.^9^

The need for children to access psychological support has increased during the pandemic. This is particularly concerning for children with chronic health conditions as they are more likely to experience psychosocial difficulties. Diabetes patients are two to three times more likely to have depression and young people with epilepsy are at an increased risk of mental health and behavioural problems.^12,19^ Due to lockdown measures, CYP’s needs for psychological support were in many cases unmet, irrespective of condition-type. Moreover, school closures removed another potential source of psychological support.^14^

Unmet mental health needs can impair the health and wellbeing of children with chronic illness in many ways.^13^ For instance, the psychological distress experienced by people with epilepsy during the pandemic may have caused an increase in seizures and poor mental health can have a negative impact on diabetes control and blood glucose levels.^12,13,19^

This study has shown that health systems policy responses to the pandemic have significantly affected the care and health of children with chronic illness in the UK, providing evidence pointing to areas that need further research. However, there are some methodological considerations and limitations of our study that need to be taken into account. Out of the 32 articles included in the analysis only 14 of them were specifically on child chronic illness in the UK. The health conditions mainly discussed were asthma, diabetes, epilepsy, cancer and inflammatory bowel diseases and some neurodevelopmental and mental health conditions. Although there seems to be some information on projected health outcomes for children with chronic illness, and some comparisons with international studies and the adult population are also drawn, there is scarce evidence thus far on how changes in service provision affected children with chronic illness in the UK.

## Conclusion

This review has demonstrated that health systems policy responses to the pandemic had wide-reaching impacts on the delivery of, and access to, child healthcare in the UK. However, the specific assessment of the impact of service restrictions and innovative practice on the health and wellbeing of children with chronic illness in the UK is limited. Important implications about evidence-based policy can, however, be drawn from this research. The lack of sufficient evidence suggests that only a small number of studies exploring children’s health needs during the pandemic have been conducted, signifying that children’s needs have not been fairly considered in evaluating health systems policy responses to the pandemic. The paucity of research into child health does not only apply to the subject of Covid-19 but it is a general problem suggesting the potential of bias in the development of information technology and healthcare planning,^37^ and can therefore have wide-reaching policy implications. Assessing whether and how children’s needs were met during the pandemic is an important step to designing resilient and effective health systems and informing policy response to future emergencies. Future research is required to fulfil knowledge gaps regarding in access to effective diagnostic and treatment investigations and their impact on a range of paediatric patients during the pandemic. This scoping review is the first phase of work investigating the impact of the health system changes on children with chronic illness in order to inform the development of an ethical framework for how the needs of those children should be fairly considered in the pandemic context.

## Supplemental Material

sj-pdf-1-inq-10.1177_00469580221081445 – Supplemental Material for Changes in Healthcare Provision During Covid-19 and Their Impact on Children With Chronic Illness: A Scoping Review Click here for additional data file. Supplemental Material, sj-pdf-1-inq-10.1177_00469580221081445 for Changes in Healthcare Provision During Covid-19 and Their Impact on Children With Chronic Illness: A Scoping Review by Sapfo Lignou, Jenny Greenwood, Mark Sheehan, and Ingrid Wolfe in INQUIRY: The Journal of Health Care Organization, Provision, and Financing
