# Supplementary material for: Changes in Healthcare Provision During Covid-19 and Their Impact on Children With Chronic Illness: A Scoping Review
Source: Inquiry. 2022 Mar 28;59:00469580221081445. doi: 10.1177/00469580221081445 (PMC8969043; doi:10.1177/00469580221081445)
Supplement: sj-pdf-1-inq-10.1177_00469580221081445 – Supplemental Material for Changes in Healthcare Provision During Covid-19 and Their Impact on Children With Chronic Illness: A Scoping Review [file sj-pdf-1-inq-10.1177_00469580221081445.pdf]

Databas e	Query 1	Query 2	Inclusion criteria for title screening	Exclusion criteria for title screening	Inclusion criteria for abstract screening	Exclusion criteria for abstract screening
PubMed	((children) OR (child) OR (adolescent) OR (paediatric) OR (young people)) AND (("Covid-19") OR (Covid19)) AND (("health systems") OR ("health services") OR (health policy) OR (healthcare)) AND ((chronic illness) OR (diabetes) OR (asthma) OR ("complex condition") OR (cancer) OR ("heart disease") OR (epilepsy) OR (autism) OR (leukemia) OR (lymphoma) OR (disabilities)) AND (("mental health care") OR (hospital) OR ("primary care") OR (telemedicine) OR ("emergency department") OR (paediatrician) OR (clinician)) AND ((impact)	((children) OR (child) OR (adolescent) OR (paediatric) OR (young people)) AND (("Covid-19") OR (Covid19)) AND (("health services") OR (healthcare)) AND ((chronic illness) OR (diabetes) OR (asthma) OR ("complex condition") OR (cancer) OR ("heart disease") OR (epilepsy) OR (autism) OR (leukemia) OR (lymphoma) OR (disabilities)) AND (("mental health care") OR (hospital) OR ("primary care") OR (telemedicine) OR ("virtual appointment") OR ("emergency department") OR (paediatrician) OR (clinician)) AND ((impact) OR ("health outcomes") OR (perceptions)	a) if they referred to clinical guidance regarding delivery of care for a specific paediatric condition or chronic illness b) if they referred to paediatric clinical problems c) if it was unclear whether they referred to the UK context d) if it was unclear whether they concerned adult population only e) if it was unclear whether they referred to direct or indirect impact of covid-19	a) if they only concerned COVID-19 illness b) if they referred to adult care only c) if they were not relevant to the UK context d) if they concerned research/clinical trials f) if they were related to clinical training	a) if they discussed clinical outcomes for children b) if were relevant to the UK context or if it was not clear in the abstract whether they were relevant to the UK context c) if they referred to both adult and paediatric population d) if abstract was not available e) if they focused on the UK context and discussed changes in care delivery and chronic illness	a) if they discussed treatment for covid-19 or transmission and impact of Covid-19 infection on patients with chronic illness b) if they discussed impact on paediatric care in countries other than the UK c) if they discussed telemedicine strategies not related to chronic illness and paediatric patients d) if they discussed treatments/implementation of safe systems for delivery of care during the pandemic but not impact of the pandemic on care/health outcomes for children f) clinical guidelines/guidance/suggested frameworks that did not include information about child health systems/care/outcomes f) if they discussed service evaluation for adult patients g) if they discussed telemedicine/implementation of safe systems for delivery of care during the pandemic but were not related to paediatric care h) if they discussed novel triage methods for preservation of healthcare resources

	OR ("health outcomes") OR (perceptions) OR (views) OR (indications) OR ("care management") OR ("late presentation") OR ("staff redeployment ") OR ("reduced attendance") OR (delays) OR ("availability of services") OR ("accessibility of services")) Excecuted 23 June 2021	OR (views) OR (opinions) OR (satisfaction) OR (coping) OR (indications) OR (delays) OR ("availability of services") OR ("accessibility of services")) AND ((questionnaires) OR ("focus groups") OR (interviews) OR (thematic) OR (carer) OR (parental) OR (father) OR (mother) OR ("health professional"))	on children's wellbeing			
PsychInfo	((children or child or adolescent or paediatric or young people) and (Covid-19 or Covid19) and (health systems or health services or health policy or healthcare) and (chronic illness or diabetes or asthma or complex condition or cancer or heart disease or epilepsy or autism or leukemia or lymphoma or disabilities)	((children or child or adolescent or paediatric or young people) and (Covid-19 or Covid19) and (health services or healthcare) and (chronic illness or diabetes or asthma or complex condition or cancer or heart disease or epilepsy or autism or leukemia or lymphoma or disabilities) and (mental health care or hospital or primary care or telemedicine	a) if they referred to clinical guidance regarding delivery of care for a specific paediatric condition or chronic illness b) if they referred to paediatric clinical problems c) if it was unclear whether they referred to the UK context d) if it was	a) if they only concerned COVID-19 illness b) if they referred to adult care only c) if they were not relevant to the UK context d) if they concerned research/clinical trials f) if they were related to clinical training	a) if they discussed clinical outcomes for children b) if were relevant to the UK context or if it was not clear in the abstract whether they were relevant to the UK context c) if they referred to both adult and paediatric population d) if	a) if they discussed treatment for covid-19 or transmission and impact of Covid-19 infection on patients with chronic illness b) if they discussed impact on paediatric care in countries other than the UK c) if they discussed telemedicine strategies not related to chronic illness and paediatric patients d) if they discussed treatments/implementation of safe systems for delivery of care during the pandemic but not impact of the pandemic on care/health outcomes for children f) clinical guidelines/guidance/suggested frameworks that did not include information about child health systems/care/outcomes f) if they discussed service evaluation for adult patients

	and (mental health care or hospital or primary care or telemedicine or emergency department or paediatrician or clinician) and (impact or health outcomes or perceptions or views or indications or care management or late presentation or staff redeployment or reduced attendance or delays or availability of services or accessibility of services)).af.	or virtual appointment or emergency department or paediatrician or clinician) and (impact or health outcomes or perceptions or views or opinions or satisfaction or coping or indications or delays or availability of services or accessibility of services) and (questionnaires or focus groups or interviews or thematic or carer or parental or father or mother or health professional)).af.	unclear whether they concerned adult population only e) if it was unclear whether they referred to direct or indirect impact of covid-19 on children's wellbeing		abstract was not available e) if they focused on the UK context and discussed changes in care delivery and chronic illness	g) if they discussed telemedicine/implementation of safe systems for delivery of care during the pandemic but were not related to paediatric care h) if they discussed novel triage methods for preservation of healthcare resources
